# Susceptibility of *Toxoplasma gondii* to Ethanolic Extract of *Tinospora crispa* in Vero Cells

**DOI:** 10.1155/2019/2916547

**Published:** 2019-11-18

**Authors:** Alhassan Abdullahi Sharif, Ngah Zasmy Unyah, Norshariza Nordin, Rusliza Basir, Mohammed Nasiru Wana, Ashraf Alapid Ahmad, Tijjani Mustapha, Roslaini Abd. Majid

**Affiliations:** ^1^Universiti Putra Malaysia, Serdang 43400, Selangor, Malaysia; ^2^Faculty of Clinical Sciences, College of Health Sciences, Bayero University Kano, PMB 3011, Kano, Nigeria; ^3^Department of Biological Sciences, Faculty of Sciences, Abubakar Tafawa Balewa University, Bauchi, Nigeria; ^4^Department of Zoology, Faculty of Science-Alassaba, University of Gharyan, Garyan, Libya; ^5^Department of Biological Sciences, Faculty of Science, Yobe State University, PMB 1104, Damaturu, Nigeria

## Abstract

**Background:**

Toxoplasmosis remains widely distributed globally and is one of the major neglected parasitic zoonotic infections. The infection is still endemic in most parts of the world due to poor control as well as challenges of the currently used medications which can be overcome by using natural products. This study evaluated the effect of ethanolic extract from the stem of *Tinospora crispa* (EETC) on host cell invasion and intracellular replication of *Toxoplasma gondii*.

**Method:**

The stem powder of *T*. *crispa* was soaked in absolute ethanol for 72 hours. The resulting ethanolic extract was screened for the presence of phytochemicals. Vero cells monolayer in 96-well plate was infected with RH strain of *T*. *gondii* and treated with concentrations of the EETC, Veratrine alkaloid, and clindamycin ranging from 1.56 to 200 *μ*g/mL. MTT assay was conducted after 24 hours to evaluate the cytotoxicity and antiparasitic activities of the EETC. Four and 24 hours treatment models were adapted to assess the infection index and intracellular proliferation of *T*.

**Results:**

The study revealed that the EETC had no cytotoxic effects on Vero cells with IC_50_ = 179 *μ*g/mL, as compared to clindamycin (IC_50_ = 116.5 *μ*g/mL) and Veratrine alkaloid (IC_50_ = 60.4 *μ*g/mL). The EETC had good anti-toxoplasma activities with IC_50_ = 6.31 *μ*g/mL in comparison with clindamycin (IC_50_ = 8.33 *μ*g/mL) and Veratrine alkaloid (IC_50_ = 14.25 *μ*g/mL). The EETC caused more than 70% and 80% reduction in infection index and intracellular proliferation in both treatment models, respectively.

**Conclusion:**

This *in vitro* study showed that the EETC contains promising phytochemicals effective against *T*. *gondii* and safe to the host cells.

## 1. Introduction

Toxoplasmosis is a zoonotic parasitic infection caused by an obligate intracellular protozoon, *Toxoplasma gondii*. The infection was reported to affect one-third of the world population with transient or severe manifestations, depending on immune status [1]. Because of its zoonotic origin, humans mostly acquire the infection through ingestion of tissue cyst stage buried within the tissues of other intermediate hosts such as cattle, pigs, goats, sheep, and chickens. Predatory activities between the animals also favor continuous transmission of the parasite without passing the sexual life cycle stage in the definitive host, the feline cats [2]. Mother-to-fetus transmission also occurs with associated obstetric and fetal abnormalities depending on pregnancy stage during infection [3, 4]. Environmental and anthropogenic factors have been reported to contribute in the transmission of the organism through soil, vegetables, fruits, water, and food substances [1, 5]. However, infection with the tachyzoite stage is of acute onset that manifests transiently and provokes intact immune status which helps control its spread.

Most of the drugs currently used to cure toxoplasmosis are only active on the tachyzoite and are associated with severe side effects. The drugs are usually given in combination such as pyrimethamine and sulfadiazine, sulfamethoxazole and trimethoprim, to HIV/AIDs patients as prophylaxis. The administration of spiramycine to pregnant mother also serves as prophylaxis against fetal infection [6]. Macrolides and aminoglycosides antibiotics as well as other antimalarial drugs are also considered for the treatment of toxoplasmosis. Some of these drugs were reported to have low bioavailability and poor penetration of biological barriers [6, 7]. More so, considering the biology and parasitic life style of *T*. *gondii*, the challenges such as drug intolerance, severe side effects, low efficacy, and limited targets that characterized the use of current chemotherapeutic agents may pave way for the development of drug resistance [8, 9]. Because of the setbacks that limited the use of the current chemotherapeutic agents, there is need, therefore, to search for a new antitoxoplasma agent that is effective against the parasite and safer on humans. Such new agents should be able to act on the intracellular parasite, to prevent progression to chronic stage of the infection.

*Tinospora crispa* is used traditionally to cure many ailments such as malaria, diabetes, septicemia, scabies, helminthiasis, and effective on wound healing [10]. The plant was shown to possess a wide range of biological activities such as anticancer, antimicrobial, antioxidant, immunomodulatory, anti-inflammatory, and antipyretic [11]. The antiparasitic effects of *T*. *crispa* on *Plasmodium* species [12–15], filarial worms [16], and *T*. *gondii* [17] have been reported both *in vitro* and *in vivo*. Phytochemical analysis of an extract from *T*. *crispa* revealed the presence of many compounds responsible for its pharmacological activities. These phytochemicals include the diterpenoids, furanoditerpenes, flavonoids, lignans, saponins, glycosides, sterols, and alkaloids [10, 11]. More so, several subconstituents have been identified from the major groups depending on the solvents used for isolation [18–20]. It was shown that isolated fractions of these compounds or their synthetic analogs possess antiparasitic effects as reported using alkaloids of plant origin on *T*. *gondii* [21, 22] and flavonoids on *Plasmodium falciparum*, *Cryptosporidium parvum,* and *Encephalitozoon intestinalis* [23, 24].

Light microscopic analysis of intracellular parasite after exposure to herbal extract or its isolated fraction has been shown to be useful in the assessment of drug activity [25–28]. This is important in that the intracellular proliferation of *T*. *gondii* can be monitored as to whether the drug can act on intracellular parasite to prevent disease progression or subject the tachyzoite to stage interconversion to form a chronic bradyzoite stage. Therefore, in this study, the effect of the EETC on host cell invasion and intracellular replication of the tachyzoite stage of *T*. *gondii* in Vero cell was evaluated.

## 2. Materials and Methods

### 2.1. Reagents and Compounds

Clindamycin phosphate, Penicillin/Streptomycin (P/S), 0.25% trypsin/EDTA, Veratrine alkaloid, RPMI-1640, Fetal bovine serum (FBS), Bismuth III nitrate, atropine, quercetin, andrographolide, Folin-Ciocalteus, and MTT (3-(4,5-dimethylthiazol-2-yl)-2,5-diphenyltetrazolium bromide) reagents were purchased from Sigma Aldrich, USA. Other chemicals such as dimethyl sulphoxide (DMSO), bromocresol green, and gallic acid were purchased from Biobasic, Canada, while absolute ethanol was from VWR Chemicals, France.

### 2.2. Plant Extract Preparation

The *T*. *crispa* stem was purchased from Ethno resources Sdn. Bhd. and was certified by botanist of Institute Biosains (IBS), Universiti Putra Malaysia, Serdang, Selangor, Malaysia, by comparing with deposited specimens (SK1550/07). Subsequently, the stem was processed into the powder form by the company. The extraction was conducted as described previously [29, 30] with some modifications. The stem powder (100 g) was soaked in a conical flask with 900 mL of 99.9% ethanol for 72 hours at room temperature. The mixture was filtered with Whatman No. 1 filter paper and the filtrate was evaporated to dryness under reduced pressure at 50°C with a rotary evaporator (EYELA, N-N series, Switzerland) until it yields a sticky dark brown crude extract weighing 15.08 g which was kept at 4°C for subsequent use. Twenty milligrams (20 mg) portion of the extract was used to prepare a stock of 4.5 mg/mL and kept at −20°C for the subsequent experiment. During each experiment, the stock was diluted serially with complete culture medium (RPMI-1640, 10% heat-inactivated FBS or 2% heat-inactivated FBS and 1% P/S).

### 2.3. Qualitative Phytochemical Screening

The EETC was used for the qualitative screening of some major phytochemicals such as flavonoids (Alkaline test and AlCl_3_ test), alkaloids (Dragendorff test and Wegner's test), tannins and phenolics (FeCl_3_ test), terpenoids (Salkowski's test), saponins (Foam test), glycosides (Libermann's test and Keller–Kiliani's test). Atropine, quercetin, gallic acid, and andrographolide were used for alkaloids, flavonoids, tannins and phenolics, and terpenoids, respectively, as the positive standard for comparison [31–34].

### 2.4. Quantitative Phytochemical Screening

The UV/Vis spectrophotometer (Beckman Coulter, DU® 730, Life Science, USA) was used to quantify the phytochemical compounds (alkaloids, flavonoids, terpenoids, tannins, and phenolics) each using standard (atropine, quercetin, andrographolide, and gallic acid), respectively. Various concentrations of each standard were prepared. Absorbance from the EETC and standard preparations were recorded against blank for alkaloids and atropine at 470 nm, flavonoids and quercetin at 510 nm, terpenoids and andrographolide at 538 nm, tannins and gallic acid at 725 nm, and phenolics and gallic acid at 550 nm. Standard curves were constructed using absorbance against concentration, and estimation of the compound concentration was calculated as the equivalent of the standard per dry weight extract [35, 36].

### 2.5. Cell Culture and Parasite Maintenance

Vero cell line used in this study was obtained from a cryopreserved stock that was prepared originally from ATTC (ATTC® CCL-81™) donated from the Virology Laboratory, Faculty of Medicine and Health Sciences, Universiti Putra Malaysia. The Vero cells were routinely maintained in culture using complete growth medium (cRPMI) consisting of (RPMI-1640 supplemented with 10% heat inactivated-FBS (hi-FBS) and 1% Penicillin/Streptomycin (1% P/S) in 25 cm^2^ flasks. The cells were routinely incubated in 5% CO_2_ incubator at 37°C for the experiment. Tachyzoite of *T*. *gondii* RH strain (ATCC 50174) was provided by the Parasitology Laboratory of the Department of Medical Microbiology and Parasitology, Faculty of Medicine and Health Sciences, Universiti Putra Malaysia. The tachyzoite was routinely grown in Vero cell monolayer in tachyzoite complete growth medium (tcRPMI) consisting of RPMI 1640 supplemented with 2% hi-FBS and 1% P/S) in 5% CO_2_ incubator at 37°C. After 72 hours, the culture fluid containing the tachyzoite from the flasks was centrifuged at 1500 ×*g* for 10 minutes. The pellet containing tachyzoite and infected Vero cells was further purified by passing through a 27G needle five times and then filtered through 3 *μ*m Millipore filter as previously described [25]. The resulting suspension containing pure tachyzoite was further centrifuged again. The pellet containing the tachyzoite was resuspended in RPMI 1640 medium and counted using trypan blue with Neubauer chamber to determine the appropriate number for *in vitro* experiment while some were used for reinfection of Vero cell monolayer for a continuous propagation of the parasite.

### 2.6. Cytotoxicity Evaluation

The viability of Vero cells was evaluated using MTT assay [17] after exposure to the extract. Briefly, Vero cells were grown in 25 cm^3^ flask until confluence using cRPMI. 96-well plates were used to seed the cells at a density of 5 × 10^3^ cells/well in 100 *μ*L of cRPMI and kept in 5% CO_2_ incubator at 37°C for 24 hours. The cells were washed with 1x PBS after 24 hours and then treated with various concentrations (1.56 to 200 *μ*g/mL) of the EETC, Veratrine alkaloid, and clindamycin in triplicate and incubated at 37°C and 5% CO_2_. Clindamycin and cRPMI + 0.01% DMSO served as positive and negative controls, respectively. The cRPMI was aspirated after 24 hours and 20 *μ*L of 5 mg/mL phosphate-buffered saline (PBS) MTT solution was added to each well. After 4 hours the solution was replaced with 100 *μ*L of DMSO in each well to solubilize the blue formazan. The plate was shaken briefly, and absorbance was recorded at 540 nm with a microplate reader (Dynex, Magellan Bioscience). The percentage growth inhibition was calculated based on the formula below [17]:

(1) $$\begin{array}{c} \text{GI}=\frac{\text{average absorbance of control wells}-\text{average absorbance of treated wells}\times 100\%}{\text{average absorbance of control wells}}. \end{array}$$

### 2.7. *In vitro* Antiparasitic Assay

After reaching confluence, Vero cells monolayer seeded in 96-well plates at a density of 5 × 10^4^ cells/mL were infected with tachyzoite (parasite : cell ratio of 5 : 1, in total volume of 200 *μ*L per well) and incubated for 4 hours. The infected cells were washed twice 4 hours after infection, with serum-free media to remove nonadherent parasites. Then, 100 *μ*L of tcRPMI containing different concentrations (1.56–200 *μ*g/mL) [17, 26, 30] of the EETC, Veratrine alkaloid, and clindamycin was added to each well and incubated for the next 24 hours in triplicate. Clindamycin was used as a positive control. The tcRPMI + 0.01% DMSO was used as negative control. Anti-*Toxoplasma* activity of EETC, Veratrine alkaloid, and clindamycin was evaluated using MTT assay as described above to estimate the concentration of the compounds required to inhibit *T*. *gondii*-induced cytopathic effect by 50% as previously described [37].

### 2.8. Effect of EETC on Tachyzoite Cell Invasion and Intracellular Proliferation

**Figure 1:**
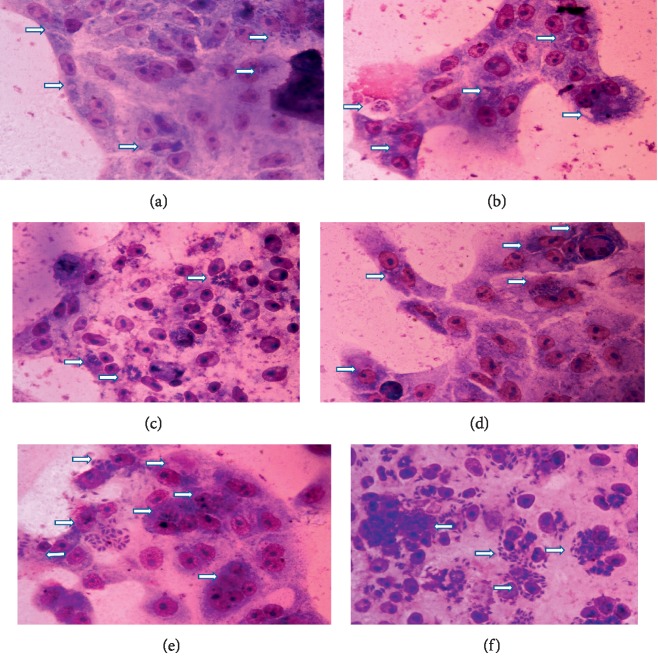
Microscopic observation of intracellular parasite at 24 hours and 48 hours postinfection treatment. Treatment of infected cells with EETC for 24 hours (a) and 48 hours (d), treatment with clindamycin for 24 hours (b) and 48 hours (e), and negative control for 24 hours (c) and 48 hours (f). The arrow indicates the parasitophorous vacuole in Vero cells where the tachyzoites proliferate. It can be observed that the normal rosette arrangement of the intracellular tachyzoite as observed in the negative control (c and f) is lost in the EETC (a and d) and to some extent in clindamycin treated group (b and e) with some empty vacuoles or vacuoles with reduced intracellular tachyzoites. Magnification ×400, scale bar 20 *μ*m.

The effect of the EETC on infection index (number of infected cells per 200 examined cells) and intracellular replication (total number of tachyzoite per 200 examined cells) of the tachyzoite was evaluated. In this regard, Vero cells were cultured on coverslips in 6-well plates (2 × 10^4^ cells/well) in complete growth medium and kept at 37°C and 5% CO_2_ for 24 hours. The cells were then infected with tachyzoite (1 × 10^5^, parasite : cell ratio 5 : 1/well) and kept at 37°C and 5% CO_2_. Four hours after infection, nonadherent tachyzoites were removed by washing with PBS twice. At this stage, the infected cells and noninfected cells were grouped in two treatment models of 4 hours and 24 hours after infection (pi). For the 4 h pi, the infected cells were treated in triplicates, using concentrations of EETC (6.31 *μ*g/mL), and clindamycin (8.33 *μ*g/mL) was used as positive control, in tcRPMI. The tcRPMI + 0.01% DMSO was used as the negative control. The plates were kept in a 37°C and 5% CO_2_ incubator. All the test groups were divided into 24 and 48 hours after incubation for analysis. The 24 h pi model was conducted as described earlier in the 4 h pi model. The coverslips from each set of experimental groups were carefully transferred using forceps onto glass slides placed on a rack. The slides were air dried, fixed for 5 minutes with methanol, and then stained with Giemsa for 15 minutes [25, 27]. The stained slides were analyzed using a light microscope (NIKON-100, Nikon Eclipse 50*i*, Japan), and all the images were captured using NIS-Element D software, Japan. Out of every 200 examined cells, the number of infected cells and the number of intracellular parasites were determined for each group of the treatment models in relation to the negative control (Figure 1) [26, 28]. The percentage inhibition was calculated using the formula:

(2) $$\begin{array}{c} \%\text{ inhibition}=\frac{1-\%\text{ inhibition in treated sample }\times \;100}{\%\text{ inhibition in nontreated sample}}. \end{array}$$

### 2.9. Statistical Analysis

Nonlinear regression analysis was used to determine the 50% inhibitory concentration (IC_50_) using GraphPad Prism 5 (GraphPad Software Inc. USA) from three independent experiments performed in triplicates. Data are presented as the mean ± SEM. One-way ANOVA and Tukey's test was used to compare the activity of different experimental groups. Differences were considered significant when *P* < 0.05.

## 3. Results

### 3.1. Phytochemical Screening

**Figure 2:**
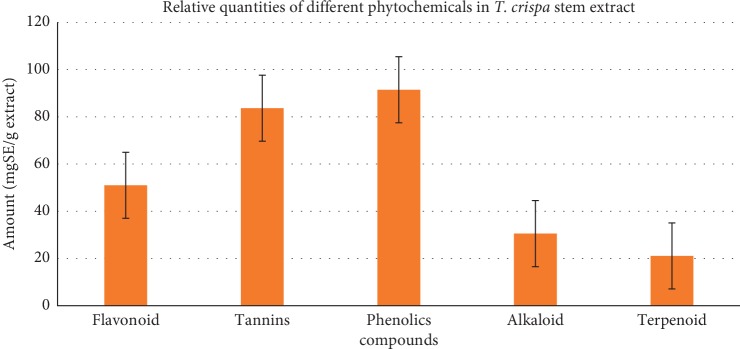
Quantity of phytochemicals estimated in ethanolic extract of stem powder of *T*. *crispa* expressed in milligram equivalent of the standard used (mgES) per gram of the dry weight extract. All data are presented as mean ± SD.

The qualitative and quantitative phytochemical screenings of the EETC are shown in Table 1 and Figure 2. Various methods were used for the screening depending on the phytochemical. The results indicated that the extract contained phytochemicals such as alkaloids, flavonoids, terpenoids, phenolics, tannins, saponins, and glycosides (Table 1). The qualitative screening was followed by quantification of each phytochemical using standard procedures.

Quantitative analysis shows that the EETC has a large quantity of phenolics of about 91.47 ± 1.2 mgGAE/g of dry extract estimated from the standard curve of gallic acid (*y* = 0.0047*x* + 0.1911, *R*^2^ = 0.995) (Figure 2). This is followed by tannins 83.67 ± 1.3 mgGAE/g estimated from standard curve of gallic acid (*y* = 0.003*x* + 0.215, *R*^2^ = 0.993). Other compounds are flavonoids, 51.0 ± 2.3 mgQE/g (*y* = 0.0036*x* + 0.1109, *R*^2^ = 0.996) and alkaloids, 30.53 ± 3.2 mgAE/g (*y* = 0.0048*x* + 0.1626, *R*^2^ = 0.994), and the least is terpenoids (21.07 ± 3.2 mgAgE/g) estimated from the standard curve of quercetin, atropine, and andrographolide, respectively. The result showed that the EETC contains a high amount of phenols followed by tannins and flavonoids.

### 3.2. Cytotoxicity and Antiparasitic Effects of EETC

**Figure 3:**
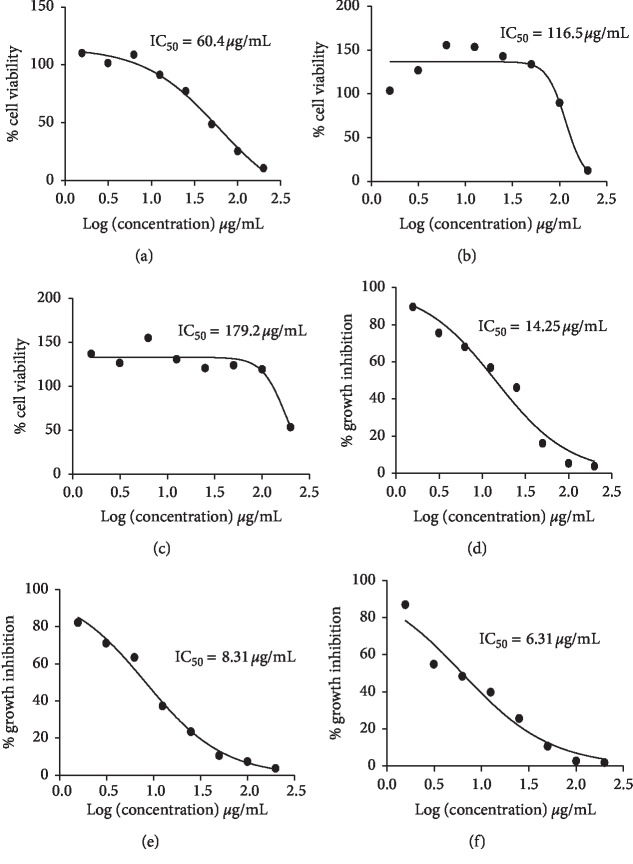
The cytotoxicity and antiparasitic graphs demonstrating 50% inhibitory concentrations. Cytotoxicity graphs of alkaloids (a), clindamycin (b), and EETC (c) each with IC_50_ = 60.4 ± 2.7 *μ*g/mL, 116.5 ± 2.3 *μ*g/mL, and 179.0 ± 1.5 *μ*g/mL, respectively, are shown. The antiparasitic activities of the alkaloids (d), clindamycin (e), and EETC (f) each with IC_50_ = 14.25 ± 0.9 *μ*g/mL, 8.33 ± 1.04 *μ*g/mL, and 6.31. ± 1.4 *μ*g/mL, respectively, are also shown. The selectivity index (SI) for alkaloids is 4.2 and clindamycin is 13.9, while that of EETC is 28.4.

The cytotoxicity and antiparasitic profile of EETC, clindamycin, and Veratrine alkaloid were evaluated (Figure 3). For cytotoxicity assay, the EETC had IC_50_ = 179 ± 1.5 *μ*g/mL, clindamycin (IC_50_ = 116.5 ± 2.3 *μ*g/mL), and Veratrine alkaloid (IC_50_ = 60.4 ± 2.7 *μ*g/mL). The effect of EETC and that of clindamycin showed that they are safe on host cells because their IC_50_ values were >100 *μ*g/ml whereas that of the alkaloids presents with moderate cytotoxicity. Antiparasitic assessment of EETC showed a high inhibitory activity on tachyzoite with IC_50_ = 6.31 ± 1.4 *μ*^g/mL^ in comparison to clindamycin (8.33 ± 1.04 *μ*g/mL) and Veratrine alkaloid (14.25 ± 0.9 *μ*g/mL). Statistical analysis showed a significant difference in the cytotoxic activities between the EETC, clindamycin, and the Veratrine alkaloid. For the antiparasitic assay, the IC_50_ obtained for alkaloid was significantly different *P* < 0.0001 from that of both clindamycin and the EETC, whereas no significant difference (*P* > 0.05) between the EETC and the clindamycin was observed. Veratrine alkaloid was shown to have a selectivity index of 4.2, clindamycin 13.9, and *T*. *crispa* 28.4. This demonstrated that the EETC is more selective , with low effect on the host cell.

### 3.3. EETC Inhibits Host Cell Invasion and Intracellular Replication

#### 3.3.1. Four Hours Postinfection Treatment Model

**Figure 4:**
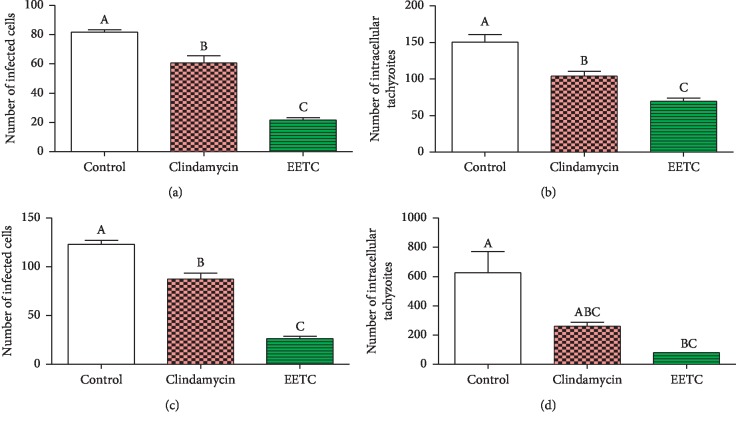
Treatment at 4 hours after infection. The infection index and intracellular replication of the parasite were analyzed at 24 hours (a, b) and 48 hours (c, d) posttreatment. The data represent the mean ± SEM of three independent studies done in triplicate. Bars with different letters differ significantly (Chart a (*P* < 0.0001), Chart b (*P* < 0.0009), Chart c (*P* < 0.0001), and Chart d (*P* < 0.01)).

After 24 hours and 48 hours treatment of infected Vero cells, cell invasion (infection index) by the tachyzoite and intracellular replication were assessed (Figure 4). For the 24 hours period, the EETC and clindamycin caused 73.47% (21.67 ± 3.0) and 25.71% (60.67 ± 8.7) reduction in infection index, respectively, in relation to the negative control (81.67 ± 3.1) (chart a, *P* < 0.001). The intracellular replication of the parasite was reduced by 53.65% (69.67 ± 7.1) for EETC and 30.81% for clindamycin (104 ± 11.5) in relation to the negative control (150.3 ± 18.2) (chart b, *P* < 0.0009). Assessment of parameters after 48 hours of infection also showed a reduction in both infection index and intracellular replication. The EETC further caused reduction of infection index by 78.59% (26.33 ± 4.16) and clindamycin by 29.00% (87.33 ± 10.8) (chart c, *P* < 0.0001) in relation to negative control (123.0 ± 7.2) and reduction in intracellular replication of 87.19% (80.33 ± 2.3) by the EETC and 58.28% (261.7 ± 45.02) by clindamycin (chart d, *P* < 0.01) in relation to negative control (627.3 ± 24.9).

#### 3.3.2. Twenty-Four Hours Postinfection Treatment Model

**Figure 5:**
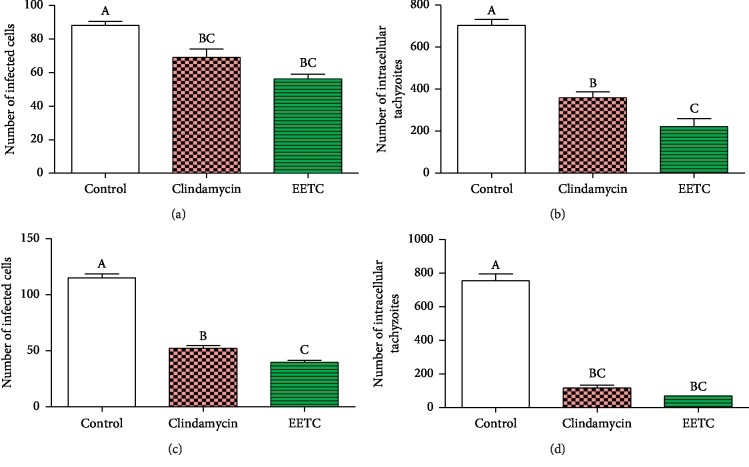
Treatment at 24 hours postinfection. The infection index and intracellular proliferation of the parasite were analyzed at 24 hours (a, b) and 48 hours (c, d) posttreatment. The data represent the mean ± SEM of three independent studies done in triplicate. Bars with different letters differ significantly (Chart a (*P* < 0.003), Chart b (*P* < 0.0001), Chart c (*P* < 0.0001), and Chart d (*P* < 0.001)).

Activities of the EETC and clindamycin were also assessed at 24 hours and 48 hours after treatment in 24 hours postinfection treatment model (Figure 5). At 24 hours posttreatment, the EETC and clindamycin caused 35.99% (56.33 ± 4.7) and 21.59% (69.0 ± 8.9) reduction in infection index, respectively, as compared to the negative control (88.0 ± 4.6) (chart a, *P* < 0.003). The intracellular replication of the parasite was reduced by 68.64% (220.7 ± 66.7) and 48.84% (360.0 ± 46.7), respectively, for EETC and clindamycin in relation to the negative control (703.7 ± 49.7) (chart b, *P* < 0.0001). Further assessment of the parameters after 48 hours of treatment also showed a reduction in both infection index and intracellular replication. The EETC and clindamycin reduced infection index by 65.6% (39.67 ± 4.2) and 54.60% (52.33 ± 4.8), respectively, in relation to the negative control (115.3 ± 6.3) (chart c, *P* < 0.0001). They also reduced intracellular replication of tachyzoite by 91.3% (69.0 ± 2.0) and 84.5% (116.7 ± 3.17) for EETC and clindamycin (chart d, *P* < 0.001), respectively, in relation to the negative control (755.0 ± 72.9) (Figure 1).

## 4. Discussion

Evidence abounds that treatment failure in the management of toxoplasmosis could be due to drug resistance [5]. Apart from their adverse effects, the drugs available for treating toxoplasmosis, most especially the first line agents, were reported to be less efficient on the parasite as well as another related genus, *Plasmodium*, because of mutation in dihydropteroate synthase (DHPS) gene. Considering the genetic diversity and the life cycle stages of *T*. *gondii* as well as variation in its susceptibility to current medication, the need for further drug development to treat toxoplasmosis is still an urgent concern in the field of parasitology and drug discovery.

Plants are still relevant as a source of new antitoxoplasma drugs. There are various studies that reported activities of plant extracts and their individual phytochemicals on apicomplexan parasites [17, 23, 38–41]. This study screened for and estimated the major phytochemicals present in the EETC such as alkaloids, flavonoids, terpenoids, phenolics, saponins, tannins, and glycosides. The finding of this study is consistent with previous studies [10, 20]. Despite the urgent need for safer drugs in the treatment of toxoplasmosis, an extract, though natural, should be analyzed on the host cell in order to have an insight into its potential cytotoxic ability. In this study, the Vero cells were used as the host cell model on the basis that they support the growth of *T*. *gondii* with good replication yield for the experimental purpose [42]. Findings in this study showed that both EETC and clindamycin have no cytotoxic effect on Vero cell with IC_50_ = 179.0 ± 1.5 *μ*g/mL and 116.5 ± 2.3 *μ*g/mL, respectively. This result is consistent with that obtained in a previous study [17]. On the contrary, the Veratrine alkaloid exhibited a moderate cytotoxic effect on the Vero cells with IC_50_ = 60.4 ± 5.7 *μ*g/mL indicating its toxicity on eukaryotic cells [43]. Alkaloid cytotoxicity on various cell lines has been reported which became the basis of their use as cancer chemotherapeutic agents [44]. The analysis indicated that the EETC is safer than clindamycin and alkaloids on the Vero cells due to its higher selectivity index. Although there was no significant difference in antiparasitic activities of the EETC and clindamycin, the result showed that the extract was more effective on the tachyzoite and safer on Vero cells than the clindamycin and Veratrine alkaloid with high selectivity index.

The antiparasitic activities of clindamycin and ethanolic extract of *T crispa* against *T*. *gondii* were shown to be effective. The ethanolic extract had an IC_50_ = 6.31 ± 1.4 *μ*g/mL and clindamycin IC_50_ = 8.33 ± 1.04 *μ*g/mL. These results are also consistent with a previous study using *T*. *crispa* extract on *T gondii* in which the antitoxoplasma effect of *T*. *crispa* using stem methanolic extract along with fractions was reported previously [17]. The study indicated 7.71 ± 1.56 *μ*g/mL as 50% effective concentration on *T*. *gondii* for the extract obtained from the stem powder as against the 6.24 ± 0.53 *μ*g/mL obtained for the positive control, clindamycin. On further fractionation, two fractions were shown to have greater effects on *T*. *gondii* than the methanolic extract, each at an effective concentration of 6.71 ± 1.95 *μ*g/mL and 6.02 ± 2.51 *μ*g/mL. The conclusion from the study did not clearly show whether that effect is due to the alkaloid content or otherwise. The present study reported an IC_50_ value of 6.31 ± 1.4 *μ*g/mL with ethanolic extract, which was shown to have a significant effect on the parasite rather than the positive control clindamycin (IC_50_ = 8.33 ± 1.04 μg/mL). However, despite the difference in IC_50_ concentrations, the result of this study further proved that the EETC contained bioactive constituents useful to develop potent drugs against *T*. *gondii*. Based on the reported activities of alkaloids on *T*. *gondii* [22, 37] and the fact that alkaloids are also one of the active constituents in *T*. *crispa* [11], this study investigated the *in vitro* effect of Veratrine alkaloid on this parasite. The antiparasitic effect of Veratrine alkaloid was at IC_50_ = 14.25 ± 0.9 *μ*g/mL which is higher than that of the EETC and has a very low selectivity index of 4.2.

Based on the selectivity index obtained, the effect of the EETC and clindamycin was investigated on the proliferation index of the tachyzoites using microscopy to determine the infection index (cell invasion) and intracellular replication. For this study, the 4 hours and 24 hours postinfection treatment models were adapted, and the EETC was seen to significantly inhibit parasite invasion, replication within the Vero cells, and egress, in relation to the negative control (Figure 1). There was a significant reduction in the number of rosettes formation as revealed by the appearance of the intracellular parasite and some infected cells were completely cleared-off the parasite (Figure 1). This particularly demonstrated the effectiveness of the bioactive compound contained in the ethanolic extract. In both treatment models, there was a reduction on both infection index and intracellular replication after 24 hours and 48 hours treatment in relation to the negative control. Both parameters were also reduced in the positive control group but to a lesser extent when compared to that of ethanolic extract. Similar studies were reported earlier [25], where *Ginkgo biloba* extract was observed to inhibit intracellular proliferation of tachyzoite in HFF cells after a short time of exposure, at a concentration that is nontoxic to the host cells. In addition, another study [45] also demonstrated the effect of an extract from *Eurycoma longifolia*, which showed clearance, and hence inhibition of the parasite proliferation in Vero cells in a concentration and time-dependent manner. It is important to note that activities of the EETC in this study are different from that of the Veratrine alkaloid and that may be due to the combined effect of the various bioactive agents present in the extract [46].

## 5. Conclusion

This study established that the EETC exhibited a significant effect on *T*. *gondii* host cell invasion and replication. This indicates that the extract contains bioactive agents to which the parasite is susceptible. Though it is important to know each of the active compounds in the EETC, the activity of the extract may by far outweigh the use of individual fractionated compound and this may be due to synergism between different bioactive molecules present in the extract. Further studies to determine the activity of the EETC using *in vivo* model and to ascertain the mechanism of action is strongly recommended so that an effective compound against this parasite can be developed.

## Figures and Tables

**Table 1:** Phytochemical analysis of the EETC.

Phytochemicals	Alkaloids	Flavonoids	Terpenoids	Phenolics	Tannins	Saponins	Glycosides
*Tinospora crispa*	++	++	++	++	++	++	++

++ = present.

## Data Availability

The datasets generated and analyzed in this study belong to the Universiti Putra Malaysia and can only be made available upon request through email to Dr Roslaini Abd. Majid (roslaini@upm.edu.my).

## Supplementary Materials

Supplementary Materials Suppl 1: Tables 1 and 2 demonstrating % growth inhibition for both cytotoxicity and antiparasitic assays, respectively. Suppl 2: the 50% inhibitory concentration results obtained from nonlinear regression analysis of the % growth inhibition from Graphad Prism software.
