# Supplementary material for: Susceptibility of Toxoplasma gondii to Ethanolic Extract of Tinospora crispa in Vero Cells
Source: Evid Based Complement Alternat Med. 2019 Nov 18;2019:2916547. doi: 10.1155/2019/2916547 (PMC6885813; doi:10.1155/2019/2916547)
Supplement: Supplementary Materials — Suppl 1: Tables 1 and 2 demonstrating % growth inhibition for both cytotoxicity and antiparasitic assays, respectively. Suppl 2: the 50% inhibitory concentration results obtained from nonlinear regression analysis of the % growth inhibition from Graphad Prism software. [file 2916547.f1.pdf]

The %growth inhibition in each treatment and control was obtained from absorbance readings (from three independent experiments) of the treated, negative control and blank wells. The % growth inhibition obtained were computed in GraphPad prism to calculate the 50% inhibitory concentration in each of the assays below using nonlinear regression analysis.

Antiparasitic assay			
	EETC	Clindamycin	Alkaloid
Concentration ug/mL	% growtth inhibition	% growtth inhibition	% growtth inhibition
200	1.9581	3.782	3.874567
100	2.7752	7.477533	5.425667
50	10.789	10.69833	16.19843
25	25.718	23.51733	46.23217
12.5	39.891	37.36217	57.02647
6.25	48.452	63.50006	68.10664
3.125	54.917	71.235	75.62217
1.56	87.061	82.27237	89.58627

Nonlin fit	EEAP
log(inhibitor) vs. normalized response -- Variable slope	
Best-fit values	
LogIC50	0.6395
HillSlope	-0.8691
IC50	4.360
Std. Error	1.43
LogIC50	0.03473
HillSlope	0.06473
95% Confidence Intervals	
LogIC50	0.5545 to 0.7244
HillSlope	-1.028 to -0.7107
IC50	3.585 to 5.302
Goodness of Fit	
Degrees of Freedom	6
R square	0.9862
Absolute Sum of Squares	63.07
Sy.x	3.242
Number of points	
Analyzed	8

Nonlin fit	Alkaloid
log(inhibitor) vs. normalized response -- Variable slope	
Best-fit values	
LogIC50	1.154
HillSlope	-1.011
IC50	14.25
Std. Error	0.90
LogIC50	0.05726
HillSlope	0.1287
95% Confidence Intervals	
LogIC50	1.014 to 1.294
HillSlope	-1.326 to -0.6959
IC50	10.32 to 19.68
Goodness of Fit	
Degrees of Freedom	6
R square	0.9677
Absolute Sum of Squares	248.6
Sy.x	6.437
Number of points	
Analyzed	8

Nonlin fit	clindamycin
log(inhibitor) vs. normalized response -- Variable slope	
Best-fit values	
LogIC50	0.9165
HillSlope	-1.049
IC50	8.252
Std. Error	1.04
LogIC50	0.02854
HillSlope	0.07028
95% Confidence Intervals	
LogIC50	0.8467 to 0.9864
HillSlope	-1.221 to -0.8769
IC50	7.026 to 9.691
Goodness of Fit	
Degrees of Freedom	6
R square	0.9907
Absolute Sum of Squares	62.83
Sy.x	3.236
Number of points	
Analyzed	8

Nonlin fit	EETC
log(inhibitor) vs. normalized response -- Variable slope	
Best-fit values	
LogIC50	0.7968
HillSlope	-0.9294
IC50	6.263
Std. Error	1.40
LogIC50	0.06406
HillSlope	0.1312
95% Confidence Intervals	
LogIC50	0.6400 to 0.9535
HillSlope	-1.250 to -0.6084
IC50	4.365 to 8.985
Goodness of Fit	
Degrees of Freedom	6
R square	0.9570
Absolute Sum of Squares	262.7
Sy.x	6.617
Number of points	
Analyzed	8

Cytotoxicity assay			
	EETC	Clindamycin	Alkaloid
Concentration ug/mL	% growth inhibition	% growth inhibition	% growth inhibition
200	53.678	12.40749	10.97759
100	119.5432	89.964	25.7424
50	124.205	133.8925	48.9711
25	120.9777	143.1653	77.452
12.5	131.0394	153.8092	91.543
6.25	155.2444	155.9238	108.901
3.125	126.7679	127.091	101.73
1.56	137.2568	103.7468	110.32

Nonlin fit	EETC
log(inhibitor) vs. normalized response -- Variable slope	
Best-fit values	
LogIC50	2.253
HillSlope	-3.448
IC50	179.2
Span	= 133.2
Std. Error	1.50
LogIC50	0.04641
HillSlope	1.448
95% Confidence Intervals	
LogIC50	2.134 to 2.373
HillSlope	-7.171 to 0.2747
IC50	136.2 to 235.9
Goodness of Fit	
Degrees of Freedom	5
R square	0.8768
Absolute Sum of Squares	753.2
Sy.x	12.27
Number of points	
Analyzed	8

Nonlin fit	EEAP
log(inhibitor) vs. normalized response -- Variable slope	
Best-fit values	
LogIC50	2.154
HillSlope	-2.654
IC50	142.5
Span	= 133.4
Std. Error	1.20
LogIC50	0.02358
HillSlope	0.3631
95% Confidence Intervals	
LogIC50	2.093 to 2.214
HillSlope	-3.588 to -1.720
IC50	124.0 to 163.9
Goodness of Fit	
Degrees of Freedom	5
R square	0.9833
Absolute Sum of Squares	138.6
Sy.x	5.265
Number of points	
Analyzed	8

Nonlin fit	clindamycin
log(inhibitor) vs. normalized response -- Variable slope	
Best-fit values	
LogIC50	2.066
HillSlope	-4.329
IC50	116.5
Span	= 136.9
Std. Error	2.30
LogIC50	0.06468
HillSlope	2.603
95% Confidence Intervals	
LogIC50	1.900 to 2.232
HillSlope	-11.02 to 2.364
IC50	79.40 to 170.8
Goodness of Fit	
Degrees of Freedom	5
R square	0.8803
Absolute Sum of Squares	1885
Sy.x	19.42
Number of points	
Analyzed	8

Nonlin fit	alkaloid
log(inhibitor) vs. normalized response -- Variable slope	
LogIC50	1.781
HillSlope	-0.7411
IC50	60.40
Span	= 192.6
Std. Error	5.70
LogIC50	0.1648
HillSlope	0.2127
95% Confidence Intervals	
LogIC50	1.357 to 2.205
HillSlope	-1.288 to -0.1942
IC50	22.77 to 160.2
Goodness of Fit	
Degrees of Freedom	5
R square	0.9483
Absolute Sum of Squares	794.7
Sy.x	12.61
Number of points	
Analyzed	8
